# Physical Characterization of Colorectal Cancer Spheroids and Evaluation of NK Cell Infiltration Through a Flow-Based Analysis

**DOI:** 10.3389/fimmu.2020.564887

**Published:** 2020-12-23

**Authors:** Azzurra Sargenti, Francesco Musmeci, Francesco Bacchi, Cecilia Delprete, Domenico Andrea Cristaldi, Federica Cannas, Simone Bonetti, Simone Pasqua, Daniele Gazzola, Delfina Costa, Federico Villa, Maria Raffaella Zocchi, Alessandro Poggi

**Affiliations:** ^1^ CellDynamics isrl, Bologna, Italy; ^2^ Laboratory of Human and General Physiology, Department of Pharmacy and Biotechnology (FaBiT), University of Bologna, Bologna, Italy; ^3^ Molecular Oncology and Angiogenesis Unit, Istituto di Ricovero e Cura a Carattere Scientifico (IRCCS) Ospedale Policlinico San Martino, Genoa, Italy; ^4^ Division of Immunology, Transplants and Infectious Diseases, Istituto di Ricovero e Cura a Carattere Scientifico (IRCCS) San Raffaele Scientific Institute, Milan, Italy

**Keywords:** colorectal cancer, spheroid, natural killer cells, mass density, microfluidics, weight, 3D cell culture

## Abstract

To improve pathogenetic studies in cancer development and reliable preclinical testing of anti-cancer treatments, three-dimensional (3D) cultures, including spheroids, have been widely recognized as more physiologically relevant *in vitro* models of in *vivo* tumor behavior. Currently, the generation of uniformly sized spheroids is still challenging: different 3D cell culture methods produce heterogeneous populations in dimensions and morphology, that may strongly influence readouts reliability correlated to tumor growth rate or antitumor natural killer (NK) cell-mediated cytotoxicity. In this context, an increasing consensus claims the integration of microfluidic technologies within 3D cell culture, as the physical characterization of tumor spheroids is unavoidably demanded to standardize protocols and assays for *in vitro* testing. In this paper, we employed a flow-based method specifically conceived to measure weight, size and focused onto mass density values of tumor spheroids. These measurements are combined with confocal and digital imaging of such samples. We tested the spheroids of four colorectal cancer (CRC) cell lines that exhibit statistically relevant differences in their physical characteristics, even though starting from the same cell seeding density. These variations are seemingly cell line-dependent and associated with the number of growing cells and the degree of spheroid compaction as well, supported by different adenosine-triphosphate contents. We also showed that this technology can estimate the NK cell killing efficacy by measuring the weight loss and diameter shrinkage of tumor spheroids, alongside with the commonly used cell viability *in vitro* test. As the activity of NK cells relies on their infiltration rate, the *in vitro* sensitivity of CRC spheroids proved to be exposure time- and cell line-dependent with direct correlation to the cell viability reduction. All these functional aspects can be measured by the system and are documented by digital image analysis. In conclusion, this flow-based method potentially paves the way towards standardization of 3D cell cultures and its early adoption in cancer research to test antitumor immune response and set up new immunotherapy strategies.

## Introduction 

In the last years, growing interest has developed in creating and evolving three-dimensional (3D) culture systems to allow more detailed studies of cancer biology and response to therapy (1–4). Indeed, 2D cultures cannot reproduce all the complex features and cell-to-cell interactions occurring at the site of lesion. On the other hand, animal models do not always reproduce the pathophysiology of human cancers, besides being very expensive and showing a negative environmental impact (5–8). Several 3D culture systems, including spheroids, have been validated by the European Union Reference Laboratories for Alternatives to Animal Testing (EURL ECVAM) as preclinical models (9–11). These systems possess many advantages over 2D cultures or animal models, such as reproducibility, the high number of replicates, systematic evaluation of multiple parameters and the possibility to set up standardized co-cultures with different cell types (12–14). In this context, we used the spheroid culture system to evaluate some aspects of the antitumor immune response exerted by a subset of T lymphocytes against colorectal cancer (CRC) cells (15). Thus, spheroids can be a reliable 3D culture system that allows the co-culture of cancer cells and effector immunocompetent cells. However, static culture conditions do not allow the evaluation of all the dynamic events occurring during tumor growth, moreover, such systems cannot fully overcome the limited distribution of oxygen or nutrients and waste removal operating *in vivo*. As a consequence, there is a growing interest for the combination of microfluidic technologies within 3D cultures, including tumor spheroids, to reproduce more faithfully the real tissue microenvironment that undergoes multiple chemical and mechanical challenges, eventually leading to changes in physical parameters (16–19).

Physical features of a tumor include spatial cell organization, external mechanical stimuli, extracellular matrix architecture and stiffness. Mechanobiological modifications, such as active stretch and tension, are other important functional aspects that may condition tumor cell growth and drug response (18, 19). Such microenvironmental aspects should be considered when using 3D culture methods, including multicellular spheroids that take advantage of the natural disposition of several epithelial tumor cells to aggregate (16, 19). Thus, the measurement of physical variations of growing tumors in vitro represents a useful tool in cancer biology, both for elucidation of the mechanisms underlying tumor development and spreading and for testing anti-cancer drugs.

In a recent paper, a linear relationship between impedance and cell number was found for some tumor cell lines and referred to proliferation rates (17). Also, cell size can be measured as volume or mass, as an indicator of cell integrity and state; more precisely, the ratio between mass and volume (i.e. mass density) can be used to distinguish between cell populations even when volume and mass do not vary (20). From this viewpoint, mass density may evidence early modifications in cellular composition that precede size and weight changes, such as organelle enlargement underlying the high metabolic rate of neoplastic cells or nuclear segmentation that occurs at the beginning of apoptotic process due, for example, to anti-cancer drugs (21). In more advanced phases of cell proliferation, mass density might also be influenced by cell number, especially during the first cellular duplications that conceivably do not alter weight and volume yet. In principle, since variations in mass density and volume occur during cell growth and are connected to changes in the cell cycle, the measurement of cell weight and mass density can provide a direct evaluation of the biological processes, underlying tumor progression and environmental changes (20, 21).

Mass density is usually indirectly monitored by software elaboration (22) of bi-dimensional images to assess the compaction degree of cells inside the spheroids, even if 2D projection might alter the real 3D structure. Some papers report mass density as “tightness” (23) “solidity” (24), “optical density” (25) and “compactness index” (26), each one presenting some measurement variations due to different chosen parameters or image quantification software. Therefore, these parameters are used to assess the effects of a specific treatment in the spheroids, such as a toxicity test, efficacy study, tumor cell resistance, and others (24).

Several methods for determining weight and mass density of cells were established with the development of nanomechanical resonator or electrokinetic microfluidic chip (20, 27–30). However, these techniques are not intended, nor proven, for reaching the average size of spheroids and organoids. Indeed, the above-mentioned systems are suitable for corpuscles ranging between nanometers to a few micrometers scale, as single cells or small samples. Moreover, data are often collected in one step, limiting sample replication and analysis repetition essential for accurate biometric studies. We have recently developed a precise and rapid technique, that enables the simultaneous determination of weight, size and mass density of sphere-like samples (31).

In the present paper, we adopted this method to further describe the physical intrinsic differences of spheroids generated from four human CRC cell lines. Furthermore, by this system, we analyzed possible variations induced by spheroid interaction with anti-cancer effector lymphocytes: natural killer (NK) cells were chosen due to their reported role in anti-tumor immunity against CRC. As effectors of natural immunity, they work without the need of recognizing tumor associated antigen by specific receptors, such as the T cell receptor, in the HLA-class I context. Thus, their action is efficient, although broadly directed towards a large panel of cancers (32–34). In this experimental setting, we recorded variations of these parameters depending on interactions between CRC spheroids and antitumor natural killer (NK) cells during the killing process. These changes are dependent on CRC infiltration by NK cells, followed by the elimination of tumor targets.

## Materials and Equipment and Methods

### Cell Cultures and Tumor Spheroid Generation

The human certified CRC cell lines HT-29 (Duke’s type B stage), HCT-15, SW620 and DLD-1, all Duke’s type C stage, were obtained from the cell bank of the Policlinico San Martino (kind gift of Blood Transfusion Centre, Dr Barbara Parodi). HCT-15 and DLD1 were derived from the same patient but present a different karyotype (35). In particular HCT-15 is quasidiploid and DLD-1 pseudiploid; SW620 derives from a metastatic site in a lymph node and is hyperdiploid, while HT-29 is hypertriploid. A detailed description of karyotype and marker designations for each cell line is available on the American Type Culture Collection website. CRC cell lines in adherent cultures were maintained in RPMI-1640 (Gibco, Life Technologies Italy, Monza) medium supplemented with 10% fetal serum (FBS, Gibco™ One Shot™ Fetal Bovine Serum, ThermoFisher Scientific Italy, Monza, Italy), penicillin/streptomycin and L-Glutamine (BioWhittaker^®^ Reagents, Lonza, Basel, Switzerland) in a humidified incubator at 37 °C with 5% CO_2_. HT-29, HCT-15, SW620, and DLD-1 were analyzed by indirect immunofluorescence and flow cytometry for the expression of the epithelial-specific antigen (ESA) and HLA-I with the specific monoclonal antibodies (mAbs) TROP1 (R&D System, Minneapolis, MN, USA) and W6/32 (American Type Culture Collection, Manassas, WA, USA) respectively, followed by Alexafluor647-goat anti-mouse anti-isotype antibody (GAM, ThermoFisher Scientific,) and cytofluorimetric analysis as reported (15). As shown in **Supplementary Figure S1**, all the cell lines were ESA positive, but only HT-29 and SW620 expressed HLA-I. CRC spheroids were generated as described (15) in flat-bottom 96-well plates (Ultra-Low attachment multiwell plates, Corning^®^Costar^®^, NY, USA) with DMEM-F12 (BioWhittaker^®^Reagents, Lonza) in serum-free medium (SFM), supplemented with EGF (Peprotech Europe, London UK) at 10ng/ml final concentration(≥1*10^6^ units/mg). Spheroids were monitored along time and dimension (perimeter, area and volume) measured on images taken with the Olympus IX70 bright field inverted microscope equipped with a CCD camera (ORCA-ER, C4742-80-12AG, Hamamatsu, Japan) by the analysis of regions of interest, defining each spheroid, with the CellSens software (version 1.12, Olympus, Tokyo, Japan) (15). Experiments were performed on day 6 of spheroids formation as at this time point all cells in culture were alive, as assessed by culturing a sample under adherent conventional conditions for 12h and subsequent identification of living cells with propidium iodide (PI, Sigma) staining (15), and the diameter of spheroids was about 100 to 200 μm. Some spheroid samples were disrupted in Ca^2+^Mg^2+^ free PBS and cells stained with the anti-ICAM1 14D12D2 (15) or the anti-PVR (DNAM1 ligand mAb MA5-13490, Invitrogen Thermo Fisher), the anti-MIC-A mAb M2032B5 (clone 12, Sino Biologicals Inc., Beijing, China) and the anti-ULPBs mAbs (anti-huULBP1 M295, anti-huULBP2 M311 and anti-huULBP3 M551) kindly provided by Amgen (Seattle, WA, M.T.A. n.200309766-001). The expression of these molecules on the CRC cell lines is shown in **Supplementary Figure S2A**. The Fc chimeras (soluble receptors fused with the Fc of human immunoglobulins): Fc-NKG2D and Fc-DNAM1 were purchased from R&D System (Minneapolis, MN, USA) and used on CRC cell lines in immunofluorescence assay followed by Alexafluor647 goat anti-human antiserum (Life Technologies). At least 5,000 cells/sample were run on a CyAn ADP cytofluorimeter (Beckman-Coulter Italia, Milan, Italy) and results analyzed with the Summit 4.3 software. The reactivity of Fc-NKG2D and Fc-DNAM1chimeras is depicted in **Supplementary Figure S2B**.

### Measurement of Weight, Diameter, and Mass Density of Spheroids

The adopted method is based on tracking the sample’s motion, when free-falling into a vertical flow-channel while the flow is at rest, to calculate its terminal velocity. This is achieved by using customized software that relies on a specific physical method, combined with detailed statistical analyses (31). Briefly, as shown in **Supplementary Figure S3**, the device is composed of a fluidic-core chip, equipped with a bright-field imaging setup, a peristaltic pump, a temperature sensor and a flow-circuit for the introduction and elimination of samples. Furthermore, the software assigns a circular reference to each image of the falling sample, allowing the extrapolation of the final radius from the physical calculation, as the average of the maximum radii obtained from each repetition. The terminal velocity is whereas calculated from the vertical position of the falling sample, and for each repetition a linear regression plot is derived. During the entire analysis different internal data control are performed: (i) initial visual screening of the operator; (ii) circular reference assignment; (iii) measurements repetition; (iv) calculation of the average radius; (v) linear regression plot analysis; and (vi) statistical validation with outliers elimination. All these check points insure the conformity of the analyzed samples in term of shape and size. CRC spheroids were fixed with PFA 4% overnight at 4°C, resuspended in 3.5mL of Dulbecco’s phosphate-buffered saline (DPBS), 1X w/o Ca^2+^& Mg^2+^(Corning® Life Sciences) at low concentration (<200 spheroids/mL), transferred in a centrifuge conical tube and then analyzed according to the previous protocol performed by Cristaldi et al. for biological samples (31). A minimum of 10 single spheroids was analyzed for every test condition and values were extrapolated from at least 10 repetitions. The same procedure was applied to CRC spheroids exposed to NK cells.The fixation protocol had no effect on mass density, as no statistical difference was observed in live and fixed spheroids of the 4 CRC cell lines (not shown). In particular, no statistical differences were found in weight and diameter variations in fixed or unfixed HT-29 and HCT-15 spheroids, while weight changes in SW620 (+53%) and DLD-1 (−49%), as well as diameter (+21% in SW620 and −14% in DLD-1). Since variations are concordant (a spheroid with a big diameter, also has a big weight, while a small spheroid in size also presents a little weight), mass density is not influenced: in fact, these spheroids show the same mass density values, as mass density normalizes the values of diameter and weight.

### Cell Viability and Cytolytic Assay by Crystal Violet Staining

NK cells were obtained from peripheral blood mononuclear cells by negative depletion using RosetteSep NK negative selection kit (StemCell Biotechnology, Vancouver, Canada) as described and cultured for 15 days with interleukin-2 (IL-2, 30IU/ml, Miltenyi Biotech, Milan, Italy) (34). NK cell population used in functional assays was >97% CD3 negative CD56 positive (34) (not shown in this paper). The number of CRC cells in a culture well was determined measuring the ATP spheroid content in that well, referred to the ATP content of the same cell line at a known cell concentration in suspension. The optimal amount of NK cells to detect the cytotoxic effect, determined by crystal violet assay or by image analysis, was 0.75x10^5^ cells/well and it corresponded approximately to a 1:1 effector to target (E:T) ratio (15). Spheroids were incubated at 37°C with NK cells, for 6h or 24h; then cytolytic activity was evaluated with the Crystal Violet Cell Cytotoxicity Assay Kit (Biovision, Milpitas, CA 95035 USA). CRC spheroids, alone or co-cultured with NK cells, at the indicated time points (6h or 24h), were transferred in conventional adherent plates and after further 24h were washed to remove NK cells.,The presence of residual NK cells was verified by bright field microscopy, based on dimension and morphology, before staining adherent CRC cells with crystal violet following manufacturer’s instructions. In further experiments, suspensions or adherent or CRC cell lines were challenged with NK cells at 1:1, 3:1 or 10:1 E:T ratios for 24h; under these conditions only living CRC cells remain adherent. Then, non-adherent cells were washed out, crystal violet staining was performed according to the manufacturer’s instructions and adherent cells were solubilized. The amount of crystal violet proportional to the number of living cells was measured with the VICTORX5 multilabel plate reader (Perkin Elmer, Milan, Italy) at the optical density (O.D.) of 595nm and referred to O.D._595_ of CRC cell samples without NK cells (15). Results are expressed as the percentage of living cells compared to CRC spheroids without NK cells.

### Measurement of Adenosine-Triphosphate (ATP)

Intracellular ATP was determined using the CellTiter-Glo® Luminescent Cell Viability Kit (Promega Italia Srl, Milan, Italy), following manufacturer’s instruction, using the luciferase reaction consisting in mono-oxygenation of luciferin catalyzed by luciferase in the presence of Mg^2+^, ATP and molecular oxygen. Luminescence was detected with the VICTORX5 multilabel plate reader (Perkin Elmer) expressed as relative light units (RLU) (15). Results are the mean±SD of 16 wells/spheroid cell line or samples of 20x10^3^ CRC cells in suspension for each cell line.

### Confocal Microscopy and Imaging of Spheroid Composition and Invasion

CRC spheroids were fixed with 4% PFA 5 min at 4°C, permeabilized with 1% NP40, washed with PBS and stained with 1µM Syto16 Green Fluorescent Nucleic Acid Stain (ThermoFisher). After washing, samples were seeded into a 96w Cell Imaging plate (Eppendorf AG, Hamburg, Germany) and run under the FV500 laser scanning confocal microscope. Images were taken with a 20x objective 0.40 NA, at Z points set every 8 to 10 μm, with FluoView 4.3b software (Olympus GmbH, Hamburg, Germany). In a first set of experiments, a z-stack analysis (12 sections of 8 µm for 80 to 96 µm total thickness) was performed for spheroids of each cell line in order to check their shape. Orthogonal cross-section of x-y focal planes were reconstructed and shown as x-z or y-z planes. In other experiments, spheroids were identified with the Threshold tool of the Image J software and nuclei were counted with the Multipoint Analyze Particle tool; at least 30 spheroids/cell line were analyzed on 6 Z points/spheroid; results are expressed as cell number/area. In other experiments, NK cells were stained with CFSE (1µg/ml/10^6^ cells) for 1h at 37°C; then they were washed, added to CRC spheroids at the E:T ratio of 1:1 and incubated at 37°C with NK cells for 24h. Samples were included in a Matrigel dome, seeded into a 96w Cell Imaging plate (Eppendorf AG) and run under the FV500 laser scanning confocal microscope. Other samples were fixed with 4%PFA, washed to remove NK cells adherent to the external spheroid layer and counterstained with the anti-ESA mAb TROP-1, followed by Alexafluor647-GAM (ThermoFisher Scientific). Images were taken at different Z points, set every 10μm, with a 20x objective 0.40 NA, analyzed with FluoView 4.3b software (Olympus GmbH) and shown in pseudocolor. Region of interest (ROI) designed on the inner perimeter of spheroids, were selected for cell count calculated with the Multipoint Analyze Particle tool of the Image J software on the ROI of 6 to 20 spheroids evaluated at 10 different Z positions.

### Cytofluorimetric Analysis of Spheroid Invasion

Spheroids incubated with NK cells as described in paragraph 1.5, were washed to remove free-floating NK cells. Staining with the anti-CD56 mAb C218, followed by Alexafluor488 anti-isotype-specific GAM (34) was then performed to label external NK cells, taking into consideration that mAbs can penetrate some peripheral layers of the spheroid (not shown), thus labeling also NK cells infiltrating spheroid periphery. The spheroids were then dissociated in phosphate buffer without Ca^++^/Mg^++^ and the resulting cell suspension was labeled with the anti-CD45 mAb 9.4, followed by Alexafluor647 anti-isotype-specific GAM (34) to identify all NK cells. Samples were run on a MACSQuant cytofluorimeter (Miltenyi Biotech, Gladbach, Germany) and results analyzed with the FlowJo software (Ashland, Oregon, USA), are expressed as Log green fluorescence intensity (a.u.) vs Log far-red fluorescence intensity (a.u.).

### Immunohistochemistry (IHC) and Digital Imaging

Samples of spheroids incubated with NK cells were centrifuged in 1.5ml tubes at 1000 rpm 1 min at RT, fixed with 4% PFA 1h at RT, washed with PBS and suspended in 50µl of melted agarose (2% in distilled water). After agarose polymerization, samples were dehydrated in a progressive series of ethanol, clarified in xylene and paraffin embedded. Then, 4µm thick serial sections were cut (3 sections every 15µm) and dried o.n. at 37°C. Using the Leica Bond Rx Automated Stainer (Leica Biosystems), the slides were dewaxed with Leica Bond Dewax solution. After treatment with Bond Epitope Retrieval, sections were stained with the anti-CD45 LCA mAb (2μg/mL, Ventana), or an isotypic unrelated antibody as negative control (Dako Cytomation) and reactions visualized using Leica Bond Refine Detection kit (DS9800) with diaminobenzidine (DAB) chromogen and a hematoxylin counterstain. Digital images were acquired using the Aperio ScanScope Slide program of the Aperio AT2 Scanner (Leica Biosystem, Aperio Technologies) at 20 to 40×. The number of infiltrating cells was calculated with the Image J Multipoint Analyze Particle tool on the ROI designed on the spheroid perimeter. Six spheroids/cell line were analyzed on 10 serial sections.

### Statistical Analysis

The Shapiro–Wilk test was performed on the measured outputs obtained from the biological experiments to analyze the distribution of the dataset based on skewness and/or kurtosis (36). For all the cases that resulted in a non-normal distribution, descriptive statistics box plots (Tukey method plots) were carried out for determining outliers’ values. Outliers were identified as individual points for the terminal velocity, mass density, diameter, and weight box plots. The presence of at least one outlier in one of the categories was considered sufficient to remove the related sample from the dataset. For these cases, the Shapiro–Wilk approach was reused to confirm the normal distribution. Data are presented as mean ± SEM or ±SD. Statistical analysis was performed using two-tailed unpaired Student's *t-*test. The cutoff value of significance is indicated in each figure legend.

## Results

### Characterization of the Physical Properties of CRC Spheroids

**Figure 1:**
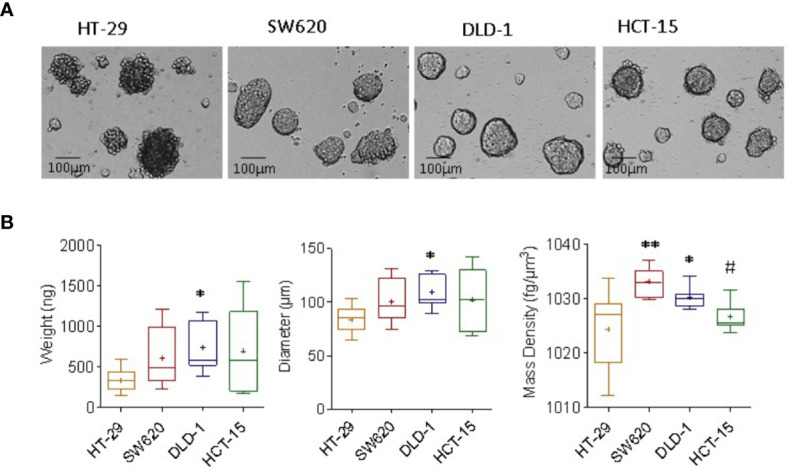
Measurement of mass density, weight and diameter of CRC spheroids. **(A)** CRC spheroids were generated with HT-29, SW620, DLD-1, and HCT-15 CRC cell lines cultured in ultra-low adherent flat-bottomed microplates and analyzed on day 6 by inverted IX70 microscope (Olympus); images were taken with 20x objective NA 0.40 (200x magnification). Bar in each panel: 100μm. **(B)** CRC spheroid samples were fixed with 4% PFA and analyzed with the flow-based system. Data are graphically depicted in box-and-whisker plots and the lines, extending from the boxes, indicate variability outside the upper and lower quartiles. Results are expressed as the weight (ng, left graph), diameter (μm, central graph) and mass density (fg/μm^3^, right graph). *p< 0.05 and **p< 0.001 vs HT-29. #p<0.05 vs DLD-1.

We analyzed tumor spheroids obtained culturing the representative CRC cells lines HT-29, SW620, HCT-15, and DLD-1 in ultra-low attachment plates, as reported (15). Ten single spheroids for each cell line were analyzed, and each spheroid values of mass density, diameter and weight, and the related standard deviation, are extrapolated from 10 repetitions. Experiments were performed on a heterogeneous population of spheroids in terms of dimension, to test the feasibility of our methods in cell aggregates having the different size ranging from 100 to 200µm diameter, as shown in **Figure 1A**. HCT-15, DLD-1, and SW620 spheroids displayed round shape with a smooth surface, while HT-29 spheroids showed irregular shape with a rough surface (**Figure 1A**). CRC spheroid samples were fixed with 4% PFA and analyzed with the flow-based system: as shown in **Figure 1B**, SW620 and DLD-1 spheroids’ weights (ng, left graph) were higher than that of HT-29. Also, their diameter (μm, central graph), calculated automatically from the images acquired during the free-fall motion, were larger than those of HT-29 spheroids. Noteworthy, the measured mass density of the sample was consistently higher in SW620 and DLD-1 than HT-29 spheroids (fg/μm^3^, right graph). As mass density represents a direct parameter to evaluate the degree of aggregate compaction, data agreed with the preliminary microscopic investigation, where HT-29 cells formed loose aggregates, instead of compact 3D tumor spheres. Weight and diameter of HCT-15 spheroids were comparable to that of SW620 and DLD-1 ones, although much more dispersed due to sample intrinsic heterogeneity (**Figure 1B**, left and central graphs); however, their mass density was similar to that of HT-29 and significantly different from that of DLD-1 (**Figure 1B**, right graph).

### Quantification of Cell Number and ATP Content Within the CRC Spheroids

**Figure 2:**
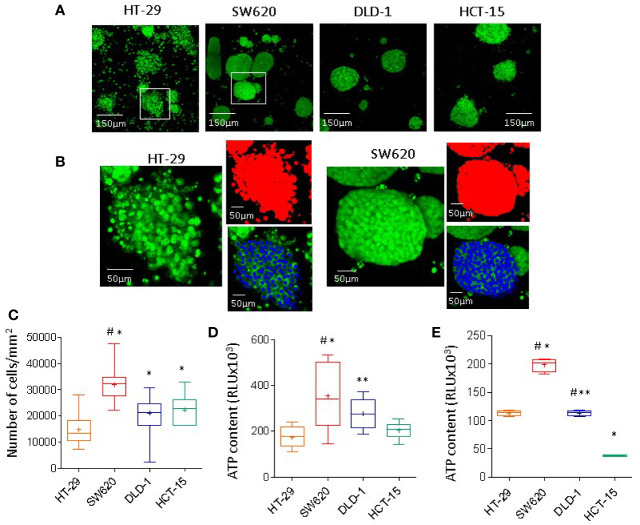
CRC spheroids cell composition and viability. **(A)** CRC spheroids of HT-29, SW620, DLD-1, or HCT-15 were stained with 1µM Syto16 Green, seeded into a 96w Cell Imaging plate (Eppendorf) and run under the FV500 laser scanning confocal microscope (200x). Images were taken at different Z points set every 10μm (one representative is shown), with FluoView 4.3b software (Olympus GmbH). **(B)** Image analysis (Image J software) of a spheroid of HT-29 (left) or SW620 (right); enlargement of the white squares in A. Spheroids identified in red pseudocolor, nuclei evidenced in blue pseudocolor. **(C)** Nuclei were counted with the multipoint and analyze particle tool; results are expressed as cell number/area (mm^2^) and are the mean±SD of 30 spheroid/cell line counted on 6 Z points/spheroid. ANOVA was performed to evaluate the differences between groups, followed by the Tukey-HSD posthoc test. *p<0.0001 vs HT-29, #p<0.0001 vs DLD-1 and HCT-15. **(D)** Intracellular ATP content measured using the CellTiter-Glo® Luminescent Cell Viability Kit (Promega). Results are expressed as relative light units (RLU) and are the mean±SD of 16 wells/spheroid cell line. Two-tailed unpaired Student’s *t*-test was performed to calculate statistical significance. *p<0.0001 vs HT-29, **p<0.001 vs HT-29, ^#^p<0.0001 vs HCT-15. **(E)** Intracellular ATP content in 20x10^3^ cells for each cell line, rescued from subconfluent (70%) cultures and kept in suspension. Results are expressed as in **(D)** and are the mean±SD of 4 wells/cell line. Two-tailed unpaired Student’s *t*-test was performed to calculate statistical significance. *p<0.0001 vs HT-29, **p<0.0001 vs SW620, ^#^p<0.0001 vs HCT-15.

To better clarify the biophysics of mass density heterogeneity, spheroids of HT-29, SW620, DLD-1, or HCT-15 cell lines underwent nuclear staining with the green Syto16 probe followed by laser scanning confocal microscope analysis. Images were taken at different Z points set every 10μm (**Figure 2A**), spheroids were identified with the threshold tool of the Image J software in red pseudocolor, while nuclei were evidenced in blue pseudocolor (**Figure 2B**) and counted with the multipoint analyze particle tool (30 spheroid/cell line counted on 6 Z points/spheroid). **Figure 2C** shows in SW620 spheroids a striking higher cell number/mm^2^ than in HT-29, DLD-1, and HCT-15 spheroids: the latter two, in turn, contain many more cells than HT-29. Intracellular ATP content was detectable in all spheroids, documenting cell viability (**Figure 2D**). The higher ATP content in SW620, and to a lesser extent in DLD-1, than in HT-29 or HCT-15 spheroids, can be referred to the higher cell number, mainly evident in SW620 spheroids (**Figure 2D** vs **Figure 2C**). Since DLD-1 and HCT-15 spheroids contained approximately the same number of cells, differences in ATP content may depend on a different metabolic state. This is also suggested by the finding that ATP values measured in cell suspensions of each cell line display differences among the four cell lines, as shown in **Figure 2E**.

### Cytotoxic Activity of NK Cells on CRC Spheroids Evaluated by Weight, Diameter, and Mass Density Measurement

**Figure 3:**
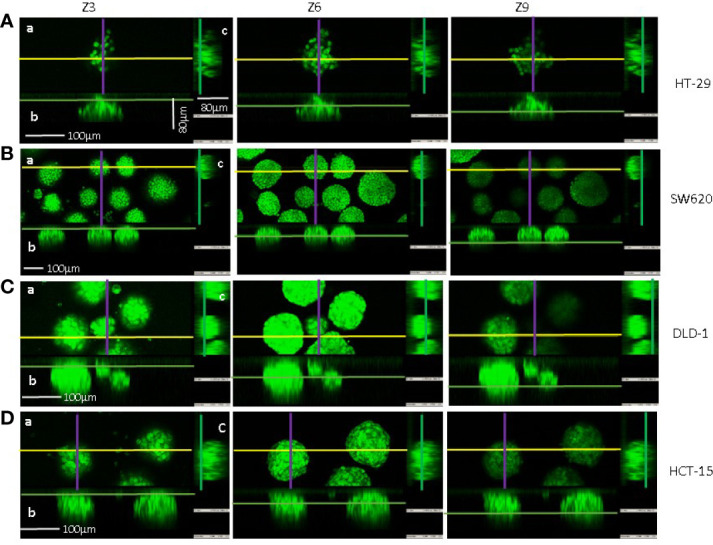
Confocal microscopy and imaging of CRC spheroid shape. Spheroids of HT-29 **(A)**, SW620 **(B)**, DLD-1 **(C)**, or HCT-15 **(D)** cell lines were stained with 1µM Syto16 Green Fluorescent Nucleic Acid Stain and run under the FV500 laser scanning confocal microscope. Images were taken with a 20x objective 0.40 NA, at Z points set every 8μm, with FluoView 4.3b software (Olympus). Orthogonal cross-section (indicated as follows: yellow for x axis, purple for y axis and green for z axis) were reconstructed from a set of a z-stack scans (12 sections for 80–96 µm total thickness: z3, z6, z9 are shown for each cell line). For each panel **(A–C)**: a) an example of x-y focal plane of the z reported in the subpanel a; b) side view of the z-stack (orthogonal x-z plane) for subpanel a); c) view in the orthogonal y-z plane for subpanel a. Bar: 100µm are indicated in panels **(A–D)** of z3.

We further planned to test the variations of physical parameters caused by the antitumor effect of NK cells and occurring during the killing process. Then, SW620, HCT-15, and DLD-1 spheroids were chosen due to their comparable weights, diameters and shape, although with different mass density. On the contrary, HT29 aggregates exhibited significantly less weight and lower, dispersed values of mass density. Confocal analysis, followed by orthogonal cross-section reconstruction from a set of a z-stack scans, showed that HT-29 spheroids were far from being spherical (example shown in **Figure 3A**) so that the flow-based system struggled to properly assign them a circular reference and, as a consequence, also the final calculated radius was affected by the samples’ irregularity. Conversely, SW620, HCT-15, and DLD-1 3D structures predominantly displayed a round shape in the orthogonal views of the z planes analyzed (z3, z6, and z9 shown in **Figures 3B–D**), indicating that they can be considered spheroids. HT29 were thereby considered less suitable models for the reliable testing of infiltrating NK cells.

**Figure 4:**
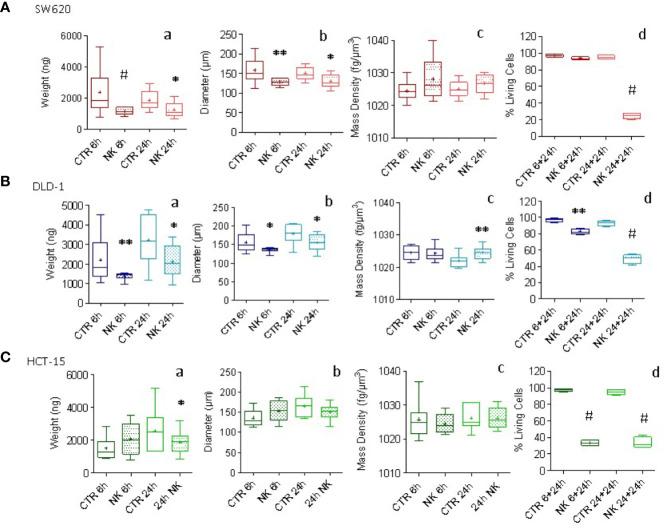
Evaluation of NK cell killing of CRC spheroids. CRC spheroids generated with SW620 **(A)**, DLD-1 **(B)** and HCT-15 **(C)** cell lines were incubated at 37°C with NK cells at the effector:target (E:T) ratio of 1:1 for 6h or 24h. Then, samples were fixed with 4% PFA and analyzed with the flow-based system. Data are graphically depicted in boxes-and-whisker plots and the lines extending from the boxes indicate variability outside the upper and lower quartiles. Results are expressed as weight (ng, a), diameter (μm, b) and mass density (fg/μm^3^, c). (graph d of **A, B, C**) Cytolytic activity evaluated in parallel samples at 6h or 24h (+additional 24h to allow living cell attachment) with the Crystal Violet Cell Cytotoxicity Assay Kit (Biovision). The amount of crystal violet proportional to the amount of living cells was measured with the VICTORX5 multilabel plate reader (Perkin Elmer) at the optical density (O.D.) of 595nm. Results are expressed as the percentage of living cells compared to CRC spheroids without NK cells. A-C: *p<0.05; **p<0.001, ^#^p<0.0005.

CRC tumor spheroids were co-incubated with NK cells at E:T ratio of 1:1 and parallel samples were analyzed for weight, diameter and mass density at 6h and 24h, while cell viability was measured with crystal violet assay. In SW620 and DLD-1 CRC spheroids, co-culture with NK cells led to a statistically significant decrease in weight (**Figures 4Aa, Ba**) and diameter (**Figures 4Ab, Bb**). In particular, SW620 weight loss was about 47% at 6h and 23% at 24h, with a decrease in the diameter of 18% and 13% respectively (**Figures 4Aa, Ab**), while DLD-1 and HCT-15 weight and diameter decreased at 24h by 34% and 13% for DLD-1 (**Figures 4Ba, Bb**) versus 23% and 7% for HCT-15 (**Figures 4Ca, Cb**). Notably, these changes were already evident at 6h, when cytotoxicity with the crystal violet assay, that is determined after 6h or 24h followed by further 24h of cell adhesion, was still undetectable or negligible (**Figures 4Ad, Bd**). Furthermore, a tendency for an increase in the mass density of SW620 and DLD-1 spheroids was observed upon co-incubation with NK cells, with different kinetics, as depicted in **Figure 4**. The tendency for an increase in SW620 mass density was already evident at 6h (**Figure 4Ac**, brown boxes and whiskers); conversely, in DLD-1 (**Figure 4Bc**, light blue boxes and whiskers) spheroids mass density significantly raised at 24h, immediately before the detection of cytotoxicity by crystal violet assay (**Figure 4Bd**). Weight and diameter of HCT-15 showed a tendency for a decrease only after 24h of co-incubation with NK cells (**Figures 4Ca and Cb**), although cytotoxicity was already evident at 6h (**Figure 4Cd**); mass density did not vary significantly, conceivably due to the small decrease in weight and diameter (**Figure 4Cc** vs **Figures 4Ca and Cb**). Thus, weight measurement can precisely reveal spheroid variations, due to NK cell cytotoxic activity, even earlier than crystal violet assay and in addition to other information such as diameter modifications and mass density changes.

When the cytolytic assay was performed with CRC cells in suspension, we observed that DLD-1 was less susceptible than SW620 and HCT-15 cell lines to NK cell-mediated lysis (**Supplementary Figure S4A**); however, this difference was not evident using the three cell lines as adherent targets (**Supplementary Figure S4B**), suggesting that cancer cell shape might influence the outcome of NK cell anti-tumor activity.

### NK Cell Infiltration of CRC Spheroids

**Figure 5:**
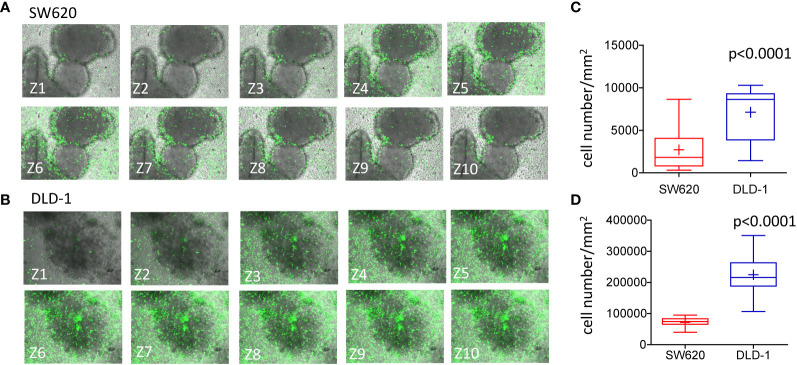
Infiltration of CRC spheroids by NK cells.I. **(A, B)**: SW620 **(A)** or DLD-1 **(B)** spheroids were seeded into a Matrigel dome in Cell Imaging plates (Eppendorf) and incubated with CFSE-labeled NK cells (E:T ratio of 1:1) for 24h. Samples were run under the FV500 confocal microscope and analyzed with FluoView 4.3b software (Olympus). Images were taken at different Z planes (Z1-Z10) every 10μm with a 20x objective NA 0.40 and shown as green CFSE^+^ NK cells merged with bright field spheroids. **(C, D)**: NK cells present in each Z plane were counted with the Multipoint Analyze Particle tool of the Image J software and plotted as the number of NK cells/mm^2^ infiltrating SW620 (red boxes and whiskers) or DLD-1 (blue boxes and whiskers) and the mean±SD of 10 Z plans of a single spheroid **(C)** or mean±SD of NK cells/mm^2^ infiltrating 20 spheroids evaluated each at 10 different Z positions **(D)**.

We further investigated whether mass density variations in SW620 and DLD-1 spheroids were associated with NK cell infiltration. To this aim, NK cells were labeled with CFSE probe (green) and NK cell entry into the spheroids was evaluated after 24h of co-culture. These two CRC spheroids were chosen since they underwent significant variations in their mass density upon 24h of interaction with NK cells. The method used for cell count applied the Multipoint and Analyze Particle tool of the Image J software to the ROI defined as single spheroids, as described in **Supplementary Figure S2**. Data were plotted as the number of infiltrating NK cells/mm^2^. **Figure 5A** shows SW620 spheroids, surrounded and progressively infiltrated by NK cells (green CFSE^+^), documented by the representative images taken at 10 different Z planes. In **Figure 5B**, NK cells invading DLD-1 spheroids are depicted, displaying an elongated shape that indicates their position inside the spheroid. A significantly higher number of NK cells could infiltrate DLD-1 (**Figures 5C, D**, blue boxes and whiskers) compared to SW620 (**Figures 5C, D**, red boxes and whiskers) spheroids, where NK cells accumulated at the periphery (**Figure 5C**: mean±SD of NK cells/mm^2^ in a single spheroid evaluated at 10 different Z positions; **Figure 5D**: mean±SD of NK cells/mm^2^ infiltrating 20 spheroids evaluated each at 10 different Z positions). These data might explain the differences in mass density detected with the flow-based system and reveal a different mode of action of NK cells to attack tumors, depending on the biological features of tumor spheroids associated with their physical properties.

**Figure 6:**
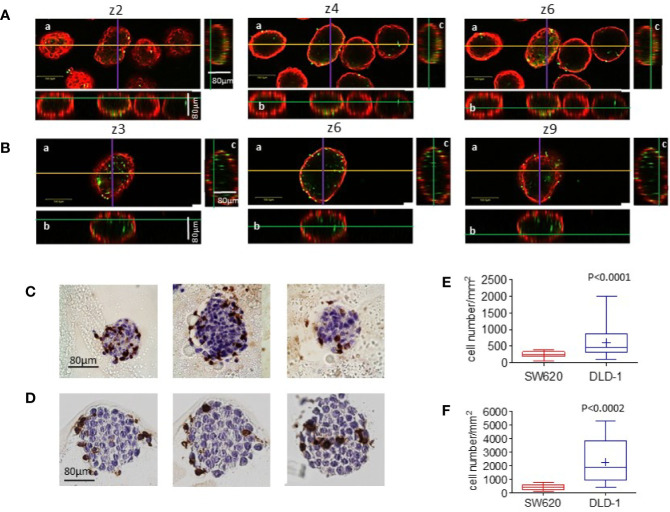
Infiltration of CRC spheroids by NK cells II. (A-B): SW620 **(A)** or DLD-1 **(B)** spheroids were seeded as in **Figure 5**, incubated with CFSE-labeled NK cells (green, E:T ratio of 1:1) for 24h, washed and counterstained with the anti-ESA mAb TROP-1, followed by Alexafluor647-GAM (red). Samples were run under the FV500 confocal microscope and analyzed with FluoView 4.3b software (Olympus). Images were taken at different Z planes (Z1-Z10) with a 20x objective NA 0.40: three representative z stack sections are shown. Orthogonal cross-section (indicated as yellow line for x axis, purple for y axis and green for z axis) were reconstructed from z-stack scans (80 µm total thickness). For each panel: a) an example of x-y focal plane of the z reported in the subpanel a; b) side view of the z-stack (orthogonal x-z plane) for subpanel a); c) view in the orthogonal y-z plane for subpanel a. Bar in subpanels a: 100µm. **(C, D)**: Other samples of SW620 **(C)** or DLD-1 **(D)** spheroids incubated with NK cells were fixed, suspended in melted agarose and paraffin embedded. Four µm thick serial sections were cut (3 sections every 15 µm) and stained with the anti-CD45 LCA mAb, visualized with DAB chromogen (brown) and a hematoxylin counterstain. Digital images were acquired using the Aperio ScanScope Slide program of the Aperio AT2 Scanner with a 40X objective. **(E, F)**: The number of infiltrating cells was calculated with the Image J Multipoint Analyze Particle tool on the ROI designed on the inner spheroid perimeter (graph E), defined on the basis of ESA staining showed in A and B, or on the visible spheroid perimeter (graph F) in the IHC stained specimens depicted in C and D. This is the main reason for the different NK cell number counted in E vs F. Six spheroids/cell line were analyzed at 10 different Z positions in **(E)** or on 10 serial sections in **(F)** Results are expressed as cell number/mm^2^. p<0.0001 **(E)** or p<0.0002 **(F)** vs SW620.

Parallel specimens of spheroids incubated with NK cells were extensively washed, to remove unbound NK cells, and counterstained with the anti-ESA mAb, followed by Alexafluor647-GAM. Samples were analyzed by confocal microscopy with the FluoView 4.3b software. **Figure 6** shows three representative z-stack images, out of 10 set every 10μm, of SW620 (A) vs DLD-1 (B) spheroid (CRC cells identified in red as ESA-positive) infiltrated by NK cells (green CFSE^+^). DLD-1 or SW620 incubated with NK cells were also included in melted agarose and paraffin embedded for IHC; serial sections were cut and stained with the anti-CD45 mAb to detect NK cells (C and D). Immunofluorescence was analyzed by Image J software on the ROI designed on the inner spheroid perimeter, to exclude NK cells confined in the external spheroid layer, defined on the basis of ESA staining; 6 spheroids/cell line were analyzed at 10 different Z positions and the number of NK cells calculated with the Image J Multipoint Analyze Particle tool. **Figure 6E** shows that the number of NK cells inside DLD-1 spheroids was significantly higher than that of NK cells infiltrating SW620 spheroids (p<0.0001). That NK cell infiltration of DLD-1 was more efficient than that of SW620 spheroids was also documented by IHC and digital imaging (**Figure 6C** vs **Figure 6D**). Image J Multipoint Analyse Particle tool was applied on the ROI defined on the visible spheroid perimeter on 10 serial sections. Also in this case, the number of infiltrating NK cells was significantly greater in DLD-1 than in SW620 spheroids (**Figure 6F**, p<0.0002).

**Figure 7:**
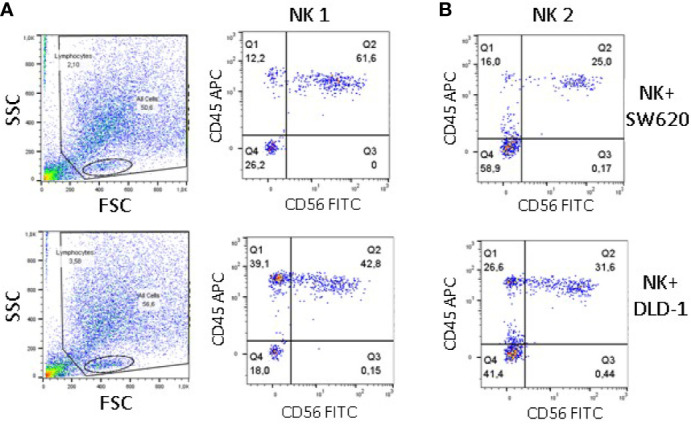
Infiltration of CRC spheroids by NK cells evaluated by cytofluorimetry. SW620 (upper panels) or DLD-1 (lower panels) spheroids were incubated with NK cells from two donors (NK1, panel **A** and NK2, panel **B**), as in **Figure 4**, washed and stained with the anti-CD56 mAb C218, followed by Alexafluor488 anti-isotype-specific GAM. After dissociation in phosphate buffer without Ca^++^/Mg^++^, the resulting cell suspension was labeled with the anti-CD45 mAb 9.4, followed by Alexafluor647 anti-isotype-specific GAM. Samples were run on a MACS Quant cytofluorimeter, 10,000 events were recorded and gating was performed on lymphocytes (panel A, left plots) Results, analyzed with the Flow Jo software, are expressed as Log green fluorescence intensity (a.u.) vs Log far-red fluorescence intensity (a.u.).In each quadrant are reported the percentage of single-positive (upper left and lower right) double-negative (lower left) or double-positive (upper right) cells.

To further verify the degree of NK cell infiltration of DLD-1 vs SW620 spheroids, staining with the anti-CD56 mAb, followed by Alexafluor488 anti-isotype-specific GAM was performed to label external NK cells. The spheroids were then dissociated and the resulting cell suspension was labeled with the anti-CD45 mAb, followed by Alexafluor647 anti-isotype-specific GAM to identify all NK cells, including those derived from the inner part of tumor spheroids. Thus, CD45 single-positive cells should identify bona fide deeply infiltrating NK cells. Samples were then analyzed by flow cytometry, gating on lymphocytes based on FSC/SSC physical parameters (**Figure 7A**, left plots). Of note, the percentage of CD45^+^CD56^-^NK cells was much higher in cell suspensions derived from DLD-1 than from SW620 spheroids (39.1% vs 12.2% NK1, **Figure 7A** right plots; 26.6% vs 16% NK2, **Figure 7B**). These findings are in line with those obtained by confocal microscopy and digital imaging analysis.

## Discussion

Alongside with the optimization of 3D cell culture methods, many efforts have been made to develop new technologies for the full characterization of these complex spherical aggregates (3, 37, 38). As regards the biophysical characterization of size and mass density, although few technical solutions have been presented for single-cell analysis, literature does not cover this aspect for 3D models of hundreds of micrometers in diameter, such as spheroids (30).

To overcome this shortage, microfluidics provides a promising technique due to its peculiar properties, including liquid handling automation, small volumes of reagents/sample required, and cost-effective fabrication. In this work, we have described a flow-based technology and the associated method for the non-invasive and accurate measure of size, weight and mass density. Although both mass and volume are important parameters for a comprehensive physical overview of the samples, mass (i.e. weight) is more fundamentally related to cell growth than is volume, thereby altering the density (39). Volume can change disproportionately to mass, thereby altering a cell’s density (39). As previously shown, this system relies on software-driven process automation that remarkably increases the ease of use of the device (30).

Tumor spheroids composed of four different CRC cell lines (HT-29, SW620, DLD-1, and HCT-15) were analyzed with this flow-based method. Interestingly, not all the CRC cell lines tested give rise to spheroids of the same shape and size, under the same experimental conditions, starting from the same number of cells seeded. Indeed, HT-29 cell spheroids appeared like loose cell aggregates where single cells can be still distinguishable. In turn, SW620 and DLD-1 spheroids displayed a smooth surface and oval (SW620) or round (DLD-1 and HCT-15) shape. This might represent a limitation, since physical measures and functional assays were performed on heterogeneous spheroids populations; nevertheless, the standardized culture conditions used allow the formation of spheroids mostly included in the range of 60 to 140 µm for all the CRC cell lines. Of note, these different characteristics can be captured, measured and evaluated by the flow-based system that can evidence distinct weight, diameter and mass density, according to the cell composition of the tumor-spheres. Although fixation, that allows sample recruitment in multicenter studies, may modify weight and diameter of spheroids in some instances, both parameters vary in the same direction so that mass density is not influenced. These findings disclose that mass density is a more reliable parameter than diameter and weight. Of note, the higher mass density detected in DLD-1 compared with HCT-15, both derived from the same patient but displaying a different karyotype (35), might indicate that the heterogeneity of clones inside the same tumor can be distinguished also on the basis of mass density measurement. In addition, the differences in spheroid mass density can be referred to the spheroid cell number. Indeed, computerized imaging, showed in SW620 spheroids a significantly higher cell number/mm^2^ than in HT-29, DLD-1, and HCT-15 spheroid: the latter two, in turn, contain a higher cell number compared to HT-29. Likewise, intracellular ATP content was not only detectable in all spheroids, as a parameter of cell viability and metabolism, but also related to cell number and mass density, determined with the flow-based system.

We could also measure the results of effector lymphocyte activity on CRC spheroids. Although controversial for many years, the role of NK cells in anti-tumor immune response is documented (31, 32, 40, 41). In CRC, the contemporary infiltration of T and NK cells is apparently linked to a better prognosis and an anti-cancer response mediated by NK cell activation has been reported in a metastatic CRC patient (42, 43). In the present flow-based system, co-culture with NK cells led to a decrease in weight and diameter of CRC spheroids, with different kinetics depending on the cell line. Indeed, SW620 and DLD-1 changes were already evident at 6 h, much earlier than cytotoxicity detection with the crystal violet assay, whereas changes in HCT-15 spheroids were evident only after 24h of co-incubation of NK cells. This might depend, at least in part, on the size, being small spheroids more susceptible to NK cell infiltration than large ones. However, the CRC spheroid populations exposed to NK cells displayed similar size variations (diameter of 80–130 µm for SW620, 90–130 µm for DLD-1 and 70–140 µm for HCT-15). Furthermore, the tendency for an increase in mass density observed at first in SW620 and later in DLD-1 spheroids, upon co-incubation with NK cells, conceivably reflects the different kinetics and degree of spheroid infiltration by NK cells. As demonstrated by confocal analysis and computerized imaging, DLD-1 spheroids are invaded by NK cells that reach in great number the inner layers and the center of the tumor by 24 h; conversely, NK lymphocytes remain in the periphery of SW620 spheroids and a few cells reach the center of the tumor mass. This behavior in invasion might explain the differences in mass density detected with the flow-based system in DLD-1 and SW620 spheroids during interaction with NK cells and evidences a different mode of action of NK cells, depending, at least in part, on the physical properties of the tumor. Also in the case of SW620 spheroids, however, the antitumor cytotoxic activity is operating, as revealed by the reduction in weight and diameter. In addition, differences in the expression of molecules involved in tumor cell killing could contribute to cancer cell sensitivity. We reported that all these CRC cell lines expressed adhesion molecules, such as ICAM1, and the ligands of NKG2D and DNAM-1 NK cell-activating receptors (35). The expression of these ligands, and the consequent reactivity of the Fc-NKG2D and Fc-DNAM1 chimeras, is maintained in CRC cell lines involved in tumor spheroid formation; this would indicate that NK cells can use most of their classic receptor-ligand systems to invade CRC spheroids and exert their anti-tumor function. On the other hand, the expression of HLA-I may be relevant to favour NK cell spheroid invasion and killing: indeed at variance with SW620, DLD-1 does not express HLA-I, that can deliver inhibitory signals to NK cells [reviewed in (33)], thus reducing their infiltrating and killing potential. Nevertheless, cancer cell shape and tumor architectural organization seem to be crucial for the outcome of NK cell anti-tumor activity; indeed the degree of cytolytic activity exerted by NK cells varies using the same tumor targets as single-cell suspensions, as adherent cells or as spheroids.

Since the 3D spheroid system is evocative of the small tumor cell clusters that may occur in the first cancer stages, a precise measurement of weight, size and density variations provide substantial information on disease progression. This is particularly relevant in CRC, where a reliable animal model is still to be defined. Moreover, with this innovative flow-based system we can measure the size and physical properties of a large number of spheroids in several replicates and different experimental conditions; this experimental setting can be useful to test new drugs or therapeutic schemes for their antitumor efficiency. Not only CRC cell lines but also primary tumor cells obtained from bioptic specimens can be used in this system, allowing the assembling of personalized precision medicine.

In conclusion, 3D spheroid models represent a reliable, reproducible and cost-effective solution that allows the evaluation and measurement of the first steps of cancer growth, taking into account the heterogeneity of tumor cells. This experimental setting also allows the evaluation of the degree and kinetics of antitumor effects exerted by immunocompetent cells; also, the system reveals any difference in the sensitivity of CRC cell types to lymphocyte effects. Potentially, the fine-tuning of the physical parameter recording could be useful in the evaluation of anti-cancer drug efficacy, including that of therapeutic antibodies and immunotherapy.

## Data Availability Statement

The raw data supporting the conclusions of this article will be made available by the authors, without undue reservation.

## Ethics Statement

The studies involving human participants were reviewed and approved by Peripheral blood mononuclear cells (PBMC) were obtained from healthy adult donor’s buffy coat upon institutional informed consent signed at the time of donation and EC approval PR163- REG2014 of the Ligurian Regional Ethics Committee. This Committee is placed at the IRCCS Ospedale Policlinico San Martino, 16132, Genoa, Italy. The patients/participants provided their written informed consent to participate in this study.

## Author Contributions

AP, MZ, and FB designed the rationale behind the work. SB, DG, DC, and SP designed and fabricated the flow-based technology. FV, AP, and MZ carried out cell culture, generation of spheroids, and performed functional assays, immunofluorescence, FACS analysis and confocal imaging. AS, FM, CD, and FC performed all the measurements with the flow-based device and statistical analysis. DC performed IHC and digital imaging analysis. AP, MZ, AS, and FB wrote sections of the manuscript. All authors contributed to the article and approved the submitted version. AP and MZ take primary responsibilities for the paper content.

## Funding

This work has been partially supported by grants from AIRC (IG-21648), Compagnia di San Paolo (ROL 32567), Ministero della Salute 5x1000 2014 and 2015 and Ricerca Corrente 2018 to AP and by the Ministry of Economic Development-AGRIFOOD PON I&C 204-2020 “Development of a technological platform for the functional testing of nutraceutical molecules”. Project Nr F/200110/01-03/X45-CUP B61B19000580008 to Cell Dynamics isrl.

## Conflict of Interest

The authors of Affiliation 1 (AS, FM, FB, DC, FC, SB, SP, and DG) are employed by Cell Dynamics isrl company. The authors declare that a Patent Application (No. 102020000006031) incorporating parts of this work has been filed. DG, SB, DC, AS, and FM are the inventors of patent No. 102020000006031.

The remaining authors declare that the research was conducted in the absence of any commercial or financial relationships that could be construed as a potential conflict of interest.

## Supplementary Material

Supplementary Figure 1 ESA and HLA-I expression on CRC cell lines. HT-29, HCT-15, SW620, and DLD-1 cell lines were analyzed by indirect immunofluorescence and flow cytometry for the expression of the epithelial-specific antigen (ESA) with the specific monoclonal antibody (mAb) TROP-1 and for and HLA-I, with the W632 mAb followed by Alexafluor647-goat anti-mouse anti-isotype antibody (GAM) (light grey histograms) (15). Samples were run on a CyAN ADP cytofluorimeter. At least ten thousand events were run and results are expressed as Log far-red fluorescence intensity (arbitrary units, a.u.) vs cell number. Dark grey histograms: negative control with Alexafluor647-GAM alone.

Supplementary Figure 2 ICAM1, NKG2DL, DNAM1L expression on CRC cell lines. **(A)** spheroid samples of the indicated CRC cell lines were disrupted in Ca^2+^Mg^2+^ free PBS and cells stained with the anti-ICAM1 14D12D2 or the anti-PVR (DNAM1 ligand, MA5-13490), the anti-MIC-A mAb M2032B5 or the anti-ULPBs mAbs (anti-huULBP1 M295, anti-huULBP2 M311 and anti-huULBP3 M551), followed by Alexafluor647 GAM. Samples were run on a CyAn ADP cytofluorimeter, results analyzed with the Summit 4.3 software and expressed as Log far red fluorescence intensity (arbitrary units, a.u.) vs number of cells. **(B)** spheroid-derived CRC cells were incubated with Fc-DNAM1 or Fc-NKG2D chimeras followed by Alexafluor647 goat anti-hu antiserum and run on the CyAn ADP cytofluorimeter. Results were analyzed and expressed as in panel **(A)**.

Supplementary Figure 3 Flow-based technology for the multiparametric physical analysis of three-dimensional biological samples. **(A)** Schematic representation of the technology system. **(B)** Front view of the field of view within the analysis channel containing the analysis medium and the 3D spheroid. Representation of the forces involved and the terminal velocity.

Supplementary Figure 4 Evaluation of NK cell killing of CRC cell lines. The SW620, DLD-1, and HCT-15 cell lines were incubated, either in suspension **(A)** or as adherent cells **(B)**, at 37°C with NK cells at the effector:target (E:T) ratio of 1:1, 3:1 or 10:1 as indicated. Cytolytic activity was evaluated at 24h with the Crystal Violet Cell Cytotoxicity Assay Kit (Biovision). The amount of crystal violet proportional to the amount of living cells was measured with the VICTORX5 multilabel plate reader (Perkin Elmer) at O.D._595_. Results are expressed as the percentage of living cells compared to CRC cells without NK cells and are the mean±SD from 8 wells with NK cells of two donors (4 wells counted for each donor). A-C: *p<0.001 vs E:T 1:1;**p<0.0001 vs E:T 1:1; #p<0.0001 vs SW620 and HCT-15.

Supplementary Figure 5 Measurement of the infiltration of CRC spheroids by NK cells. **(A)** DLD-1 spheroids were seeded into a Matrigel dome in Cell Imaging plates (Eppendorf) and incubated with CFSE-labeled NK cells (E:T ratio of 1:1) for 24h. Samples were run under the FV500 confocal microscope and analyzed with FluoView 4.3b software (Olympus). Left picture: green CFSE^+^ NK cells, with an elongated shape, indicating spheroid infiltration, merged with the bright field. White lane: ROI definition for cell count as indicated in the enlarged images of the white square (central and right). Blue points indicate infiltrating lymphocytes inside the ROI. **(B)** Images taken at different Z planes (Z1-Z10) taken every 10μm with a 20x objective. Blue points: infiltrating lymphocytes inside the ROI. The number of NK cells present in each Z plane were counted with the Multipoint Analyze Particle tool of the Image J software and plotted in the right graph as cell number/mm^2^ for each section.
