# Supplementary figures and images for: Physical Characterization of Colorectal Cancer Spheroids and Evaluation of NK Cell Infiltration Through a Flow-Based Analysis
Source: Front Immunol. 2020 Dec 23;11:564887. doi: 10.3389/fimmu.2020.564887 (PMC7786051; doi:10.3389/fimmu.2020.564887)

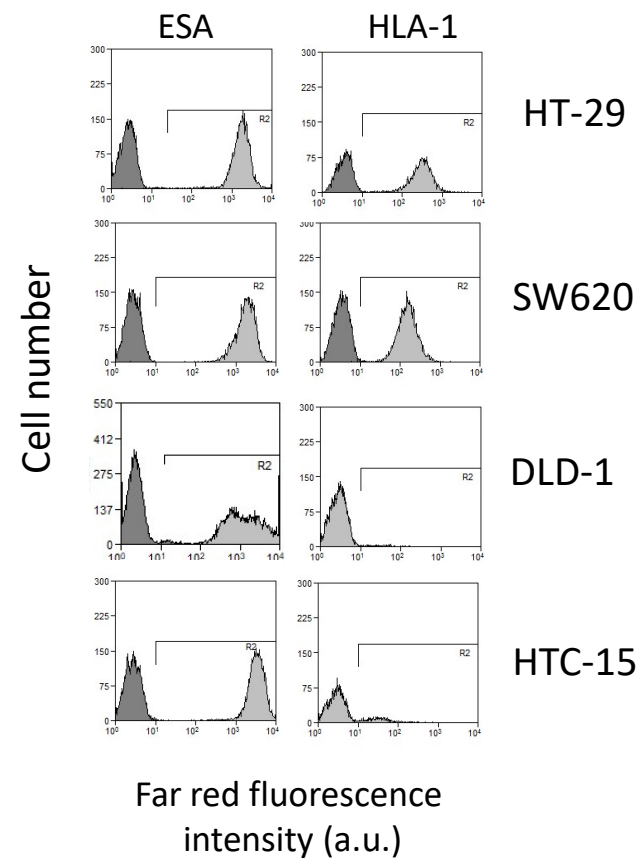

Supplement: Supplementary Figure 1 — ESA and HLA-I expression on CRC cell lines. HT-29, HCT-15, SW620, and DLD-1 cell lines were analyzed by indirect immunofluorescence and flow cytometry for the expression of the epithelial-specific antigen (ESA) with the specific monoclonal antibody (mAb) TROP-1 and for and HLA-I, with the W632 mAb followed by Alexafluor647-goat anti-mouse anti-isotype antibody (GAM) (light grey histograms) (15). Samples were run on a CyAN ADP cytofluorimeter. At least ten thousand events were run and results are expressed as Log far-red fluorescence intensity (arbitrary units, a.u.) vs cell number. Dark grey histograms: negative control with Alexafluor647-GAM alone. [file DataSheet_1.zip › Supplementary Figure 1.PDF]

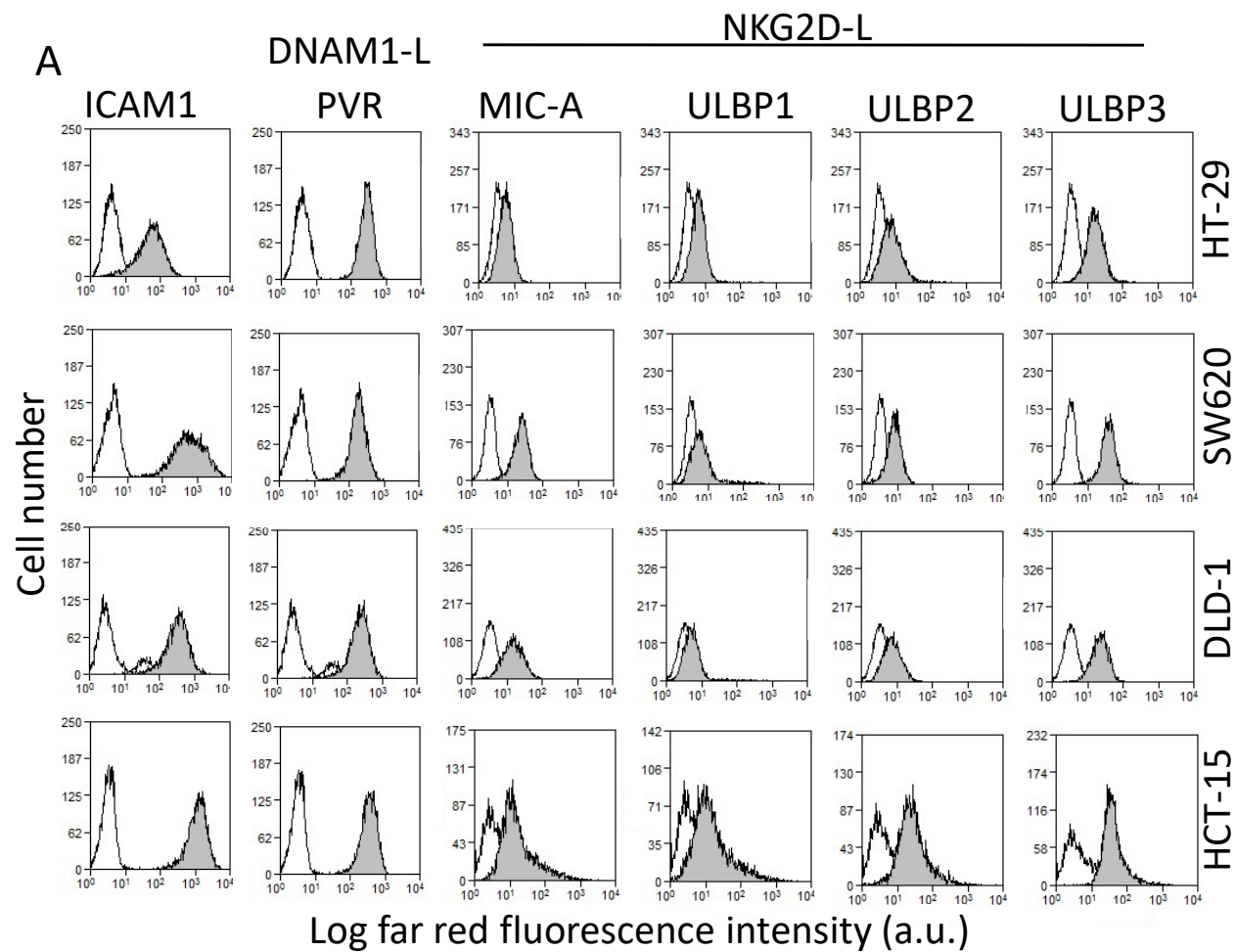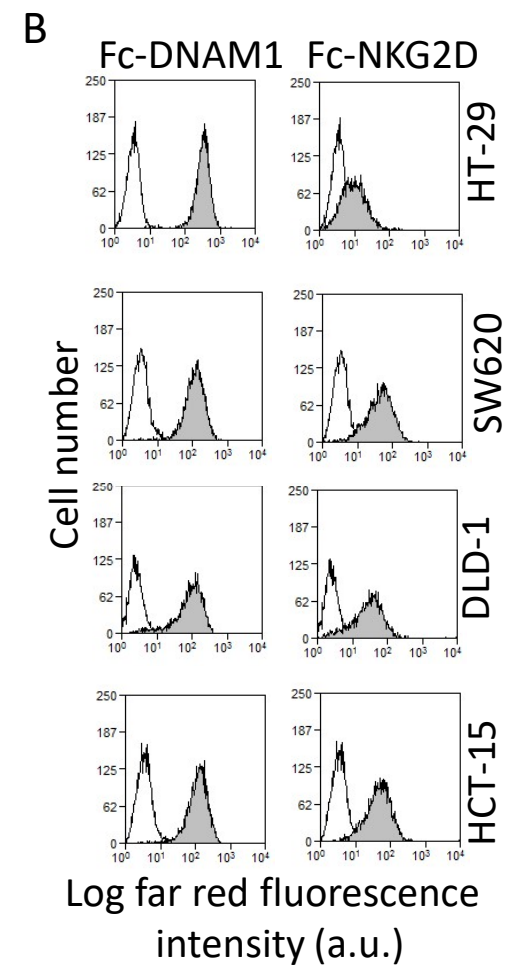

Supplement: Supplementary Figure 1 — ESA and HLA-I expression on CRC cell lines. HT-29, HCT-15, SW620, and DLD-1 cell lines were analyzed by indirect immunofluorescence and flow cytometry for the expression of the epithelial-specific antigen (ESA) with the specific monoclonal antibody (mAb) TROP-1 and for and HLA-I, with the W632 mAb followed by Alexafluor647-goat anti-mouse anti-isotype antibody (GAM) (light grey histograms) (15). Samples were run on a CyAN ADP cytofluorimeter. At least ten thousand events were run and results are expressed as Log far-red fluorescence intensity (arbitrary units, a.u.) vs cell number. Dark grey histograms: negative control with Alexafluor647-GAM alone. [file DataSheet_1.zip › Supplementary Figure 2.PDF]

A

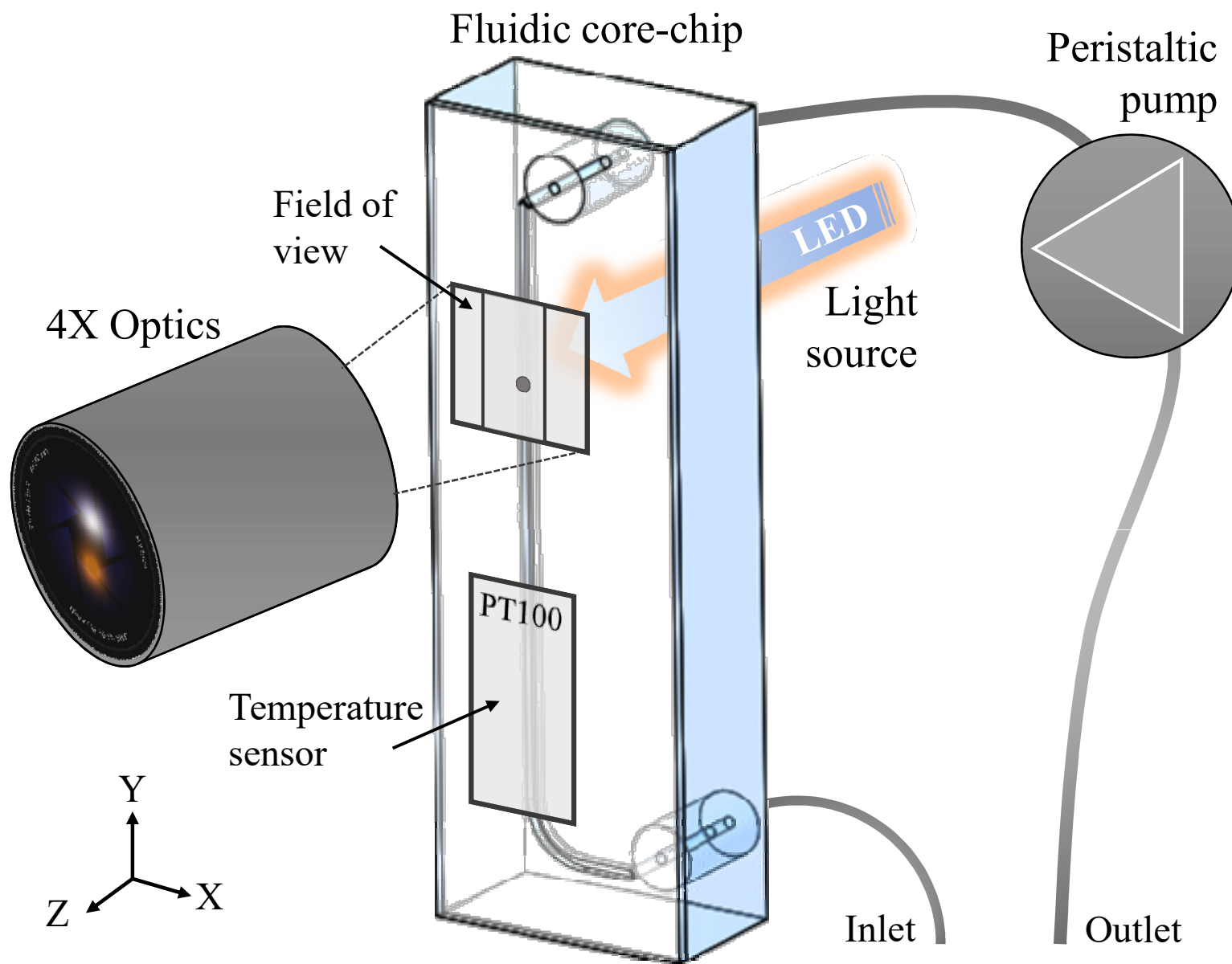

B

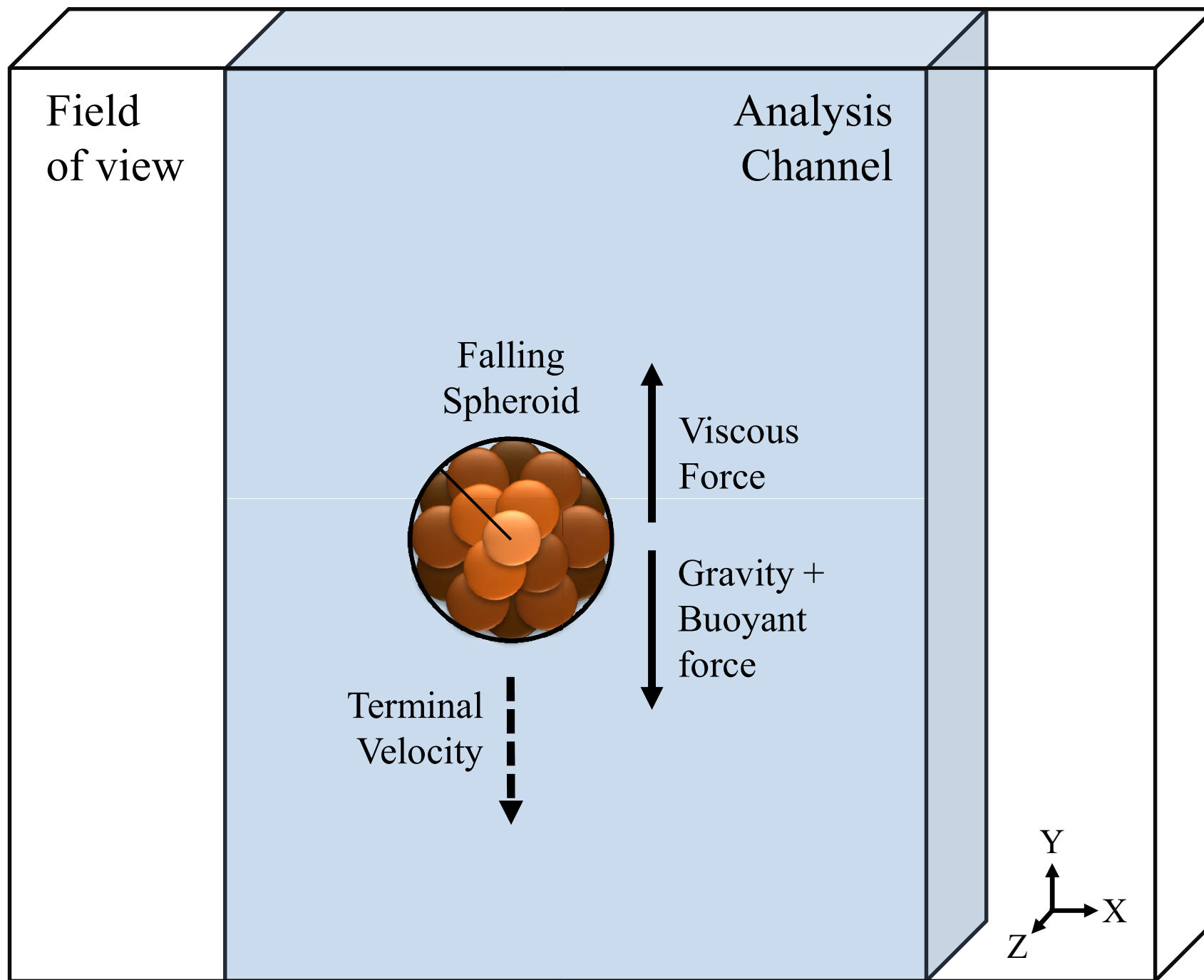

Supplement: Supplementary Figure 1 — ESA and HLA-I expression on CRC cell lines. HT-29, HCT-15, SW620, and DLD-1 cell lines were analyzed by indirect immunofluorescence and flow cytometry for the expression of the epithelial-specific antigen (ESA) with the specific monoclonal antibody (mAb) TROP-1 and for and HLA-I, with the W632 mAb followed by Alexafluor647-goat anti-mouse anti-isotype antibody (GAM) (light grey histograms) (15). Samples were run on a CyAN ADP cytofluorimeter. At least ten thousand events were run and results are expressed as Log far-red fluorescence intensity (arbitrary units, a.u.) vs cell number. Dark grey histograms: negative control with Alexafluor647-GAM alone. [file DataSheet_1.zip › Supplementary Figure 3.PDF]

A

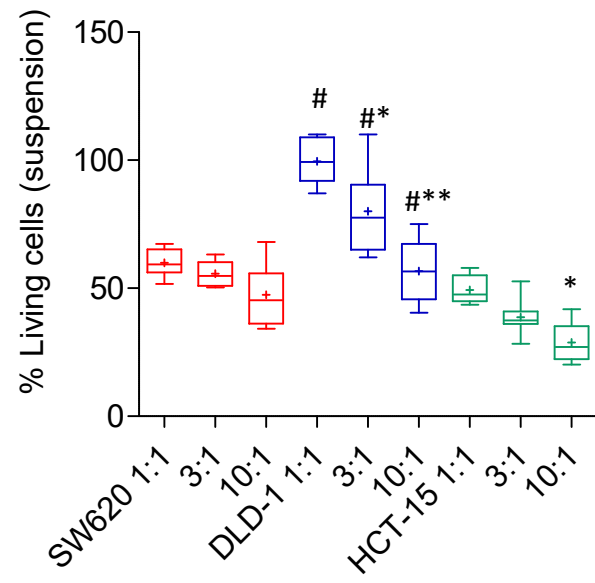

B

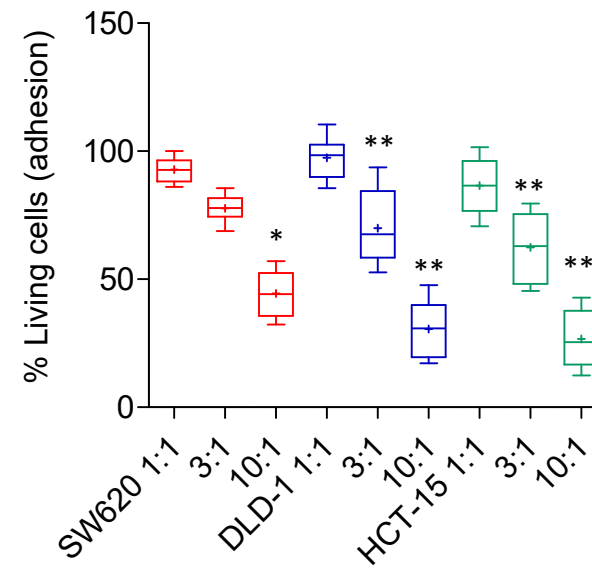

Supplement: Supplementary Figure 1 — ESA and HLA-I expression on CRC cell lines. HT-29, HCT-15, SW620, and DLD-1 cell lines were analyzed by indirect immunofluorescence and flow cytometry for the expression of the epithelial-specific antigen (ESA) with the specific monoclonal antibody (mAb) TROP-1 and for and HLA-I, with the W632 mAb followed by Alexafluor647-goat anti-mouse anti-isotype antibody (GAM) (light grey histograms) (15). Samples were run on a CyAN ADP cytofluorimeter. At least ten thousand events were run and results are expressed as Log far-red fluorescence intensity (arbitrary units, a.u.) vs cell number. Dark grey histograms: negative control with Alexafluor647-GAM alone. [file DataSheet_1.zip › Supplementary Figure 4.PDF]

# A ROI identification

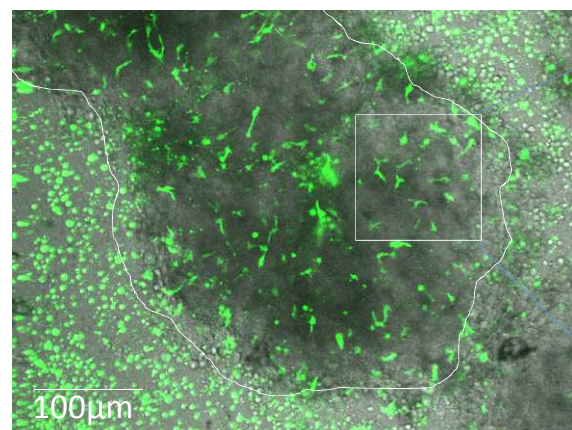

# Zoom ROI

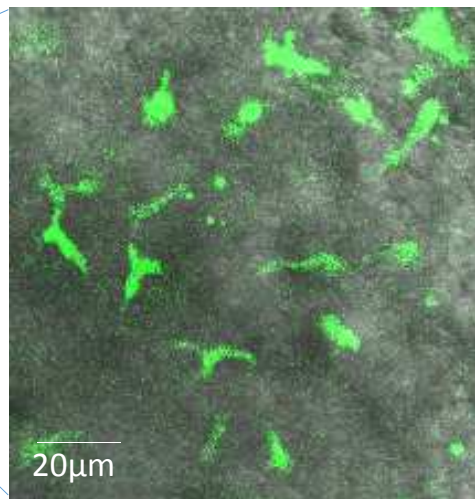

# Zoom ROI cell count

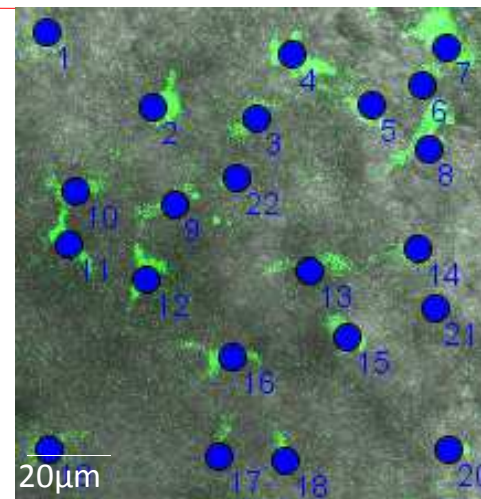

# B

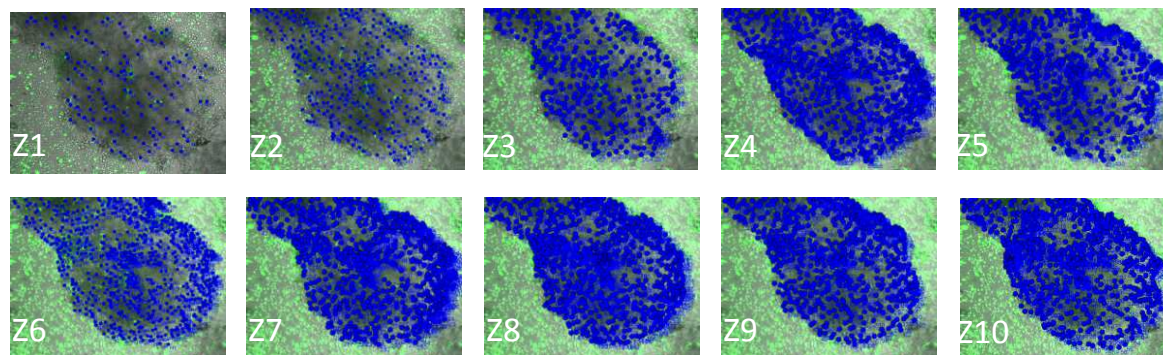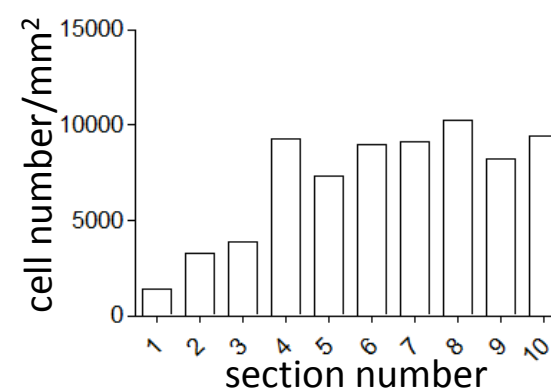

Supplement: Supplementary Figure 1 — ESA and HLA-I expression on CRC cell lines. HT-29, HCT-15, SW620, and DLD-1 cell lines were analyzed by indirect immunofluorescence and flow cytometry for the expression of the epithelial-specific antigen (ESA) with the specific monoclonal antibody (mAb) TROP-1 and for and HLA-I, with the W632 mAb followed by Alexafluor647-goat anti-mouse anti-isotype antibody (GAM) (light grey histograms) (15). Samples were run on a CyAN ADP cytofluorimeter. At least ten thousand events were run and results are expressed as Log far-red fluorescence intensity (arbitrary units, a.u.) vs cell number. Dark grey histograms: negative control with Alexafluor647-GAM alone. [file DataSheet_1.zip › Supplementary Figure 5.PDF]
